# A Dual‐Modal Memory Organic Electrochemical Transistor Implementation for Reservoir Computing

**DOI:** 10.1002/smsc.202400415

**Published:** 2024-10-16

**Authors:** Yuyang Yin, Shaocong Wang, Ruihong Weng, Na Xiao, Jianni Deng, Qian Wang, Zhongrui Wang, Paddy Kwok Leung Chan

**Affiliations:** ^1^ Department of Mechanical Engineering The University of Hong Kong Hong Kong SAR China; ^2^ Department of Electrical and Electronic Engineering The University of Hong Kong Hong Kong SAR China; ^3^ Advanced Biomedical Instrumentation Centre Hong Kong SAR China

**Keywords:** long‐term memory, neuromorphic transistors, organic electrochemical transistors, reservoir computing, short‐term memory

## Abstract

Neuromorphic computing devices offer promising solutions for next‐generation computing hardware, addressing the high throughput data processing demands of artificial intelligence applications through brain‐mimicking non‐von Neumann architecture. Herein, PEDOT:Tos/PTHF‐based organic electrochemical transistors (OECTs) with dual‐modal memory functions—both short‐term and long‐term—are demonstrated. By characterizing memory levels and relaxation times, the device has been efficiently manipulated and switched between the two modes through coupled control of pulse voltage and duration. Both short‐term and long‐term memory functions are integrated within the same device, enabling its use as artificial neurons for the reservoir unit and synapses in the readout layer to build up a reservoir computing (RC) system. The performance of the dynamic neuron and synaptic weight update are benchmarked with classification tasks on hand‐written digit images, respectively, both attaining accuracies above 90%. Furthermore, by modulating the device as both reservoir mode and synaptic mode, a full‐OECT RC system capable of distinguishing electromyography signals of hand gestures is demonstrated. These results highlight the potential of simplified, homogeneous integration of dual‐modal OECTs to form brain‐like computing hardware systems for efficient biological signal processing across a broad range of applications.

## Graphical abstract

PEDOT:Tos/PTHF‐based organic electrochemical transistor (OECT) are used as a dual‐modal memory element for neuromorphic computing. A pure‐electrical configuration strategy enables flexible short‐term memory (STM) and long‐term memory (LTM) state settings on the device. A full‐OECT reservoir computing system based on the STM and LTM devices is constructed and utilized to distinguish electromyography signals of hand gestures.

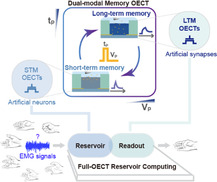

## 1 Introduction

**Figure 1:**
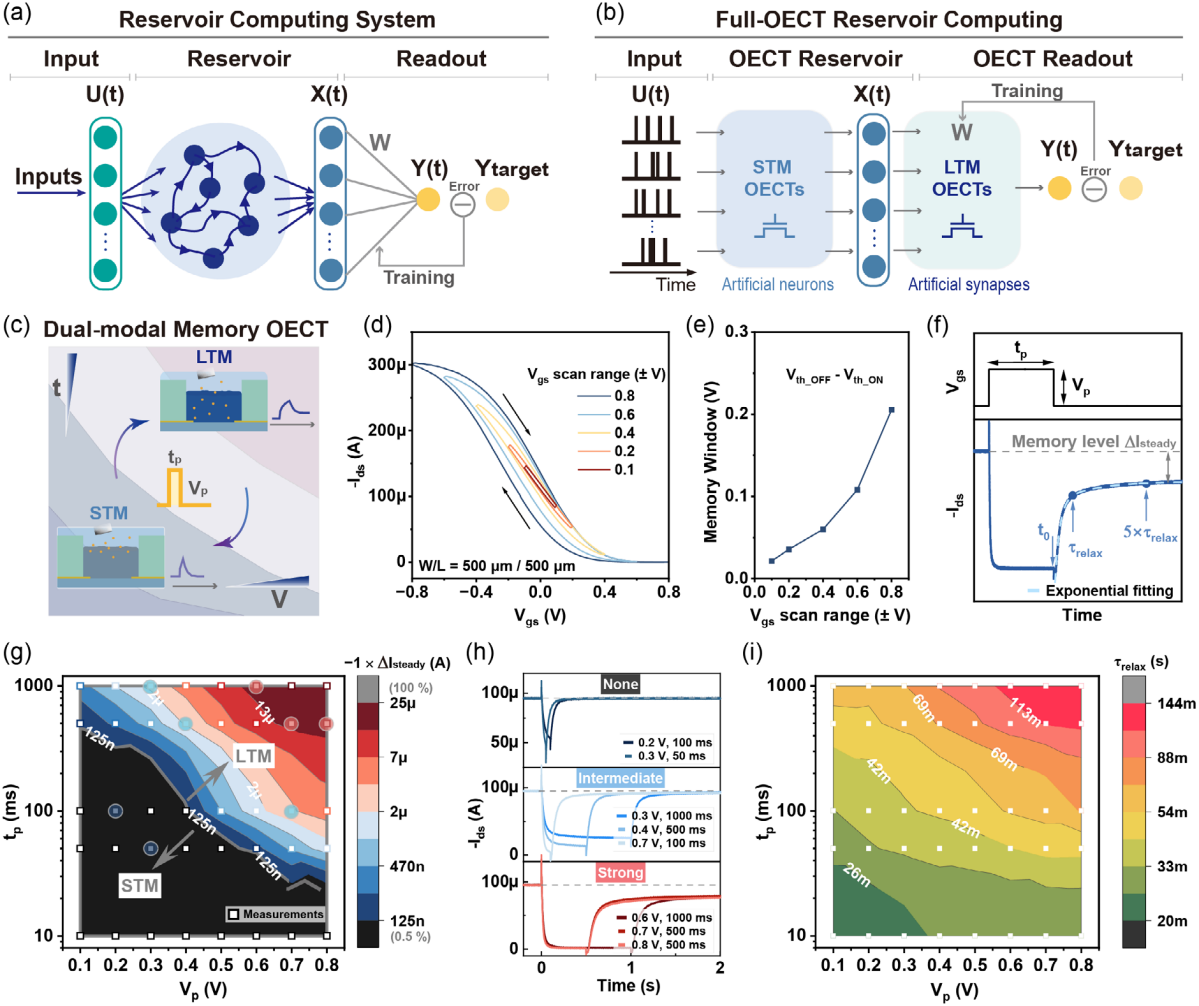
The RC hardware system concept and electrical characterizations for guiding the operation of the dual‐modal memory OECT. a) Illustration of a RC system and b) hardware implementation of full‐OECT RC. c) The concept of dual‐modal memory OECT that is configured by setting the gate pulse parameters. d) Transfer curves acquired under a series of voltage ranges of *V*_gs_ scan on the OECT with channel width and length of 500 and 500 μm. *V*_gs_ was kept at −0.2 V. e) Memory windows of transfer curves in (d) measured as the threshold voltage (*V*_th_) shift. f) Channel current response of the OECT as stimulated by a square voltage pulse. g) Contour map of memory levels given by positive gate voltage pulse inputs over a series of pulse durations and amplitudes. The contour lines are formed by interpolation between experimental data (square scatters), and the colormap presents the memory level in log scale. h) *I*_ds_ curves driven by pulses with *V*_p_–*t*_p_ combinations extracted from points around the same contour line as highlighted by circles in (g). i) Contour map of relaxing time constants *τ*_relax_ given by positive gate voltage pulse inputs over a series of pulse durations and amplitudes.

The boom in neuromorphic computing electronics provides unconventional hardware platforms for implementing artificial intelligence (AI) algorithms to process complex information, thanks to its brain‐inspired non‐von Neumann architecture.^[^ ^1^, ^2^, ^3^ ^]^ The use of neuromorphic computing for biological signal processing has become an attractive research focus, aiming to leverage miniaturized, energy‐efficient chips and low‐latency processing for in situ computing.^[^ ^4^, ^5^, ^6^, ^7^, ^8^ ^]^ Reservoir computing (RC), derived from recurrent neural network models,^[^ ^9^, ^10^, ^11^ ^]^ is an emerging computing paradigm for biosignal processing due to its advantages in handling complex temporal information. A typical RC system consists of three main components: the input layer, the reservoir layer, and the readout layer (**Figure** **1**a). The crucial unit, the reservoir, is an intrinsically nonlinear dynamic system that maps inputs ***U*** (*t*) into linearly separable reservoir states ***X*** (*t*) in feature space. This dynamic response makes it suitable for processing temporal data and solving real‐life problems, such as biosignal decoding and time‐series predictions^[^ ^12^, ^13^, ^14^, ^15^ ^]^ The separable reservoir states are then fed to the readout layer for analysis, enabling recognition with a simple linear regression network. The connectivity in the reservoir is fixed, leaving only the readout network to be trained by updating the weights ***W*** on artificial synapses based on the error between the output ***Y*** (*t*) and the ground truth ***Y***_target_. Consequently, the RC system implementation significantly simplifies the computing architecture and reduces training costs.^[^ ^11^, ^16^ ^]^

To implement the RC system into hardware, the reservoir requires physical elements that possess internal dynamics and the capability for nonlinear input projection.^[^ ^16^, ^17^ ^]^ Electronics with intrinsic nonlinearity and short‐term memory (STM) are suitable candidates for reservoir construction, such as diffusive memristors and electrochemical transistors.^[^ ^18^, ^19^, ^20^, ^21^, ^22^ ^]^ Appropriate STM ensures the dynamic behavior of the physical reservoir, allowing it to respond to the most recent features while mitigating the effects of initial conditions in temporal inputs.^[^ ^17^ ^]^ Additionally, to build artificial neural networks for readout functions, electronics with adjustable conductance/resistance are necessary to realize weight updates as artificial synapses, thereby mimicking plasticity modulation in biological synapses.^[^ ^23^ ^]^ To achieve accurate computing, nonvolatile state retention (long‐term memory or LTM), linear and symmetric conductance modulation, and low stochasticity are desired.^[^ ^2^, ^24^, ^25^ ^]^ Devices with resistance‐based or charge‐doping/trapping‐based memory are typically employed for this function.^[^ ^2^, ^3^, ^26^ ^]^ However, implementing the entire RC system on a single chip remains challenging because it involves heterogeneous devices that are responsible for each unit of the system. For example, a fully memristive reservoir computing system has been demonstrated with satisfactory performance;^[^ ^27^ ^]^ however, it still requires different device architectures with separate fabrication procedures for the reservoir and readout functions, which can increase the complexity of the overall hardware.

To improve the simplicity of building neuromorphic hardware with integrated functions, one approach is employing dual‐modal memristive devices, that is, one device is capable of both STM and LTM as configured by electrical modulations.^[^ ^28^, ^29^, ^30^, ^31^ ^]^ However, most reported devices are two‐terminal memristors, which face issues such as stochasticity in read states and higher driving voltage up to a few volts. Organic electrochemical transistors (OECTs),^[^ ^32^ ^]^ a class of three‐terminal electronic devices with subvolt operation voltages in biocompatible environments, are a high potential candidate.^[^ ^3^, ^5^, ^33^ ^]^ They have been exploited as potential building blocks of neuromorphic computing hardware, supporting both reservoir function,^[^ ^21^, ^22^ ^]^ and artificial synapse applications.^[^ ^34^, ^35^, ^36^ ^]^ The three‐terminal structure allows for simultaneous programming and reading, reducing the stochasticity of state extraction and enabling low‐latency processing, which is advantageous for online information decoding.^[^ ^37^ ^]^ Therefore, exploring strategies to fully leverage the memory functions of OECT devices to construct RC hardware for real‐life applications is highly desired.

In our previous work, we have reported a nonvolatile OECT by introducing poly(tetrahydrofuran) (PTHF) into poly(3,4‐ethylenedioxythiophene):tosylate (PEDOT:Tos) as the active layer of the transistor channel.^[^ ^38^ ^]^ The LTM character of the device can be obtained by a write bias at 0.8 V or less while the memory state can be retained for longer than 3 h. Furthermore, under the same number of consecutive gate voltage pulses, we also revealed that the memory transistor can transit from STM to LTM by varying the composition ratio of the PTHF and we also demonstrated the device potentials in associative learning circuit. The stability of the nonvolatile states and controllable transition between STM and LTM inspire a further implementation of specific machine learning models. In this work, instead of alternating the channel composition, we exploited purely electrical switching of the memory OECT between STM and LTM and applied the device in these states as the physical reservoir and artificial synapse, thus a full‐OECT RC system (Figure 1b). We first explored the dependence of memory effect and transient dynamics of the PEDOT:Tos/80% PTHF OECT device on electrical input conditions (voltage and bias duration) and used the relationships observed to guide the flexible switching of the device between STM and LTM regimes for the dual‐modal memory utility (Figure 1c). We then tested the response and performance of the device at reservoir mode and synaptic mode, respectively, with benchmarking handwriting classification tasks. Finally, a full‐OECT RC system was constructed by applying the dual‐modal states of one device, with which the electromyography (EMG) signal decoding for classifications of different hand gestures was successfully demonstrated. Our dual‐modal memory OECT is a building block for efficient neuromorphic computing. Along with the intrinsic subvolt operation and biological compatibility of OECTs, it paves the way for future exploitation of edge computing hardware for real‐life applications, especially on biosignal processing.

## 2 Results and Discussion

### 2.1 Device Configurations and Memory Effect Tuning of the OECT

The memory OECT comprising a PEDOT:Tos/PTHF channel was fabricated following the previously reported procedure as depicted in the Experimental Section. From our previous investigations, the composition of PTHF at 80% provides the desired adjustable nonvolatile memory effects.^[^ ^38^ ^]^ In the standard OECTs, the gate voltage (*V*_gs_) regulates the doping level in the semiconductor channel and the source–drain voltage (*V*_ds_) reads the source–drain current (*I*_ds_) flowing through the channel. In our memory OECTs, the *V*_ds_ was fixed at −0.2 V and *V*_gs_ was applied to control the device's memory state. The transfer curves show a clear adjustable clockwise hysteresis loop when varied *V*_gs_ are sweeping from negative to positive (on to off) and then positive to negative (off to on) to complete the loop (Figure 1d). The memory windows of the hysteresis are measured as the shift of the threshold voltage (*V*_th_) between the two transfer curves segment. As shown in Figure 1e, the memory window increases 9.7‐fold from 0.021 to 0.205 V when the scanning range of *V*_gs_ increases from 0.1 to 0.8 V. The relationship between electrical pulses and the memory state of the OECT is further explored by changing the programming gate voltage pulse duration (*t*_p_) and amplitude (*V*_p_). Figure 1f shows the typical *I*_ds_ response of a memory OECT under a gate pulse. With the response curve, the steady‐state memory level and transient response rate can be extracted. The memory level incurred by each programming pulse is defined as the difference of the steady‐state *I*_ds_ after and before the pulse input, i.e., Δ*I*_steady_, as marked in Figure 1f, and details are explained in Note S1, Supporting Information. It is worth pointing out that one can erase the memory in the device by applying an opposite polarity pulse with the same *t*_p_ and *V*_p_ (Figure S1a, Supporting Information). The *I*_ds_ curves under a series of pulse conditions (*V*_p_ = 0.1–0.8 V, *t*_p_ = 10–1000 ms) are shown in Figure S1, Supporting Information. Figure 1g presents the extracted memory levels given positive pulses in contour maps with the combined effect of *V*_p_ and *t*_p_. The colormap expresses the normalized degrees of memory level to the maximum, and we define a boundary below which the device is classified as operated in a STM (black region) while above tends to be a LTM one (Note S1, Supporting Information). The overall gradient of memory level increases along with the diagonal with a positive slope, showing the switching from STM to LTM states as controlled by pulse strength, i.e., longer pulse time and higher pulse voltage. This contour plot will serve as a reference for the design of the programming pulse scheme for the device. In Figure 1h, selected *I*_ds_ response curves under different contour line regions in Figure 1g are plotted in three panels based on their memory level group. It clearly shows different *V*_p_–*t*_p_ combinations would lead to a similar memory level. Rather than adjusting the memory effect solely by *V*_p_ or *t*_p_, this map allows quick screening of a desired *V*_p_–*t*_p_ combination for a target memory level, which is particularly important for practical applications when there are any physical boundaries such as low operating voltage or high‐speed switches required. One can not only configure such a memory OECT device as STM or LTM for multimodal neuromorphic computing applications, but also can flexibly apply the multidegree memory level for the diversity of building blocks in neuromorphic hardware.

### 2.2 Transient Dynamics of the Memory OECT

It is critical to study the dynamics of the device response to stimuli, which govern the operational time and determine the timeframe design of programming pulses. After removing the bias in the gate voltage pulse input at *t*_0_ as shown in Figure 1i, the current change of the OECT will decrease toward the original level with a relaxation time constant *τ*_relax_, which quantitively reflects the transient dynamic of the device. The *τ*_relax_ was estimated by double‐phase exponential fitting (Experimental Section and Figure S3, Supporting Information). Most of the relaxation happens within one *τ*_relax_ and it approaches to the final steady‐state value after five *τ*_relax_. Given by pulses with varied parameters, *τ*_relax_ shows an exponential correlation with *V*_p_ and power‐law relation with *t*_p_ (Figure S4a,b, Supporting Information), implying a much slower relaxing from charge injection under high *V*_p_ or long *t*_p_ conditions than under low *V*_p_ or short *t*_p_. The contour plot in Figure 1i shows the coupling effect of *V*_p_ and *t*_p_ on *τ*_relax_. Based on the information in Figure 1g,i, one can set a proper *V*_p_–*t*_p_ combination as the operating condition of the memory OECT to adjust the *τ*_relax_ while achieving the desired STM and LTM effect. Besides, OECTs with different channel dimensions can provide a diversity of device dynamics, which may benefit the application. Here, the *τ*_relax_ of two types of devices with relatively long and short channels, i.e., channel width (*W*)/channel length (*L*) are 500 μm/500 μm and 500 μm/10 μm, respectively, is compared in Figure S4c,d, Supporting Information. The devices with shorter channels would lead to lower *τ*_relax_ under lower *V*_p_–*t*_p_ conditions (e.g., 0.1–0.3 V, 10 ms) but show higher *τ*_relax_ when applying higher *V*_p_–*t*_p_, e.g., up to 1.5‐fold relative to the *τ*_relax_ of the longer channel devices under 0.6–0.8 V, 1000 ms pulse.

### 2.3 Physical Reservoir Based on STM States of the Memory OECT

**Figure 2:**
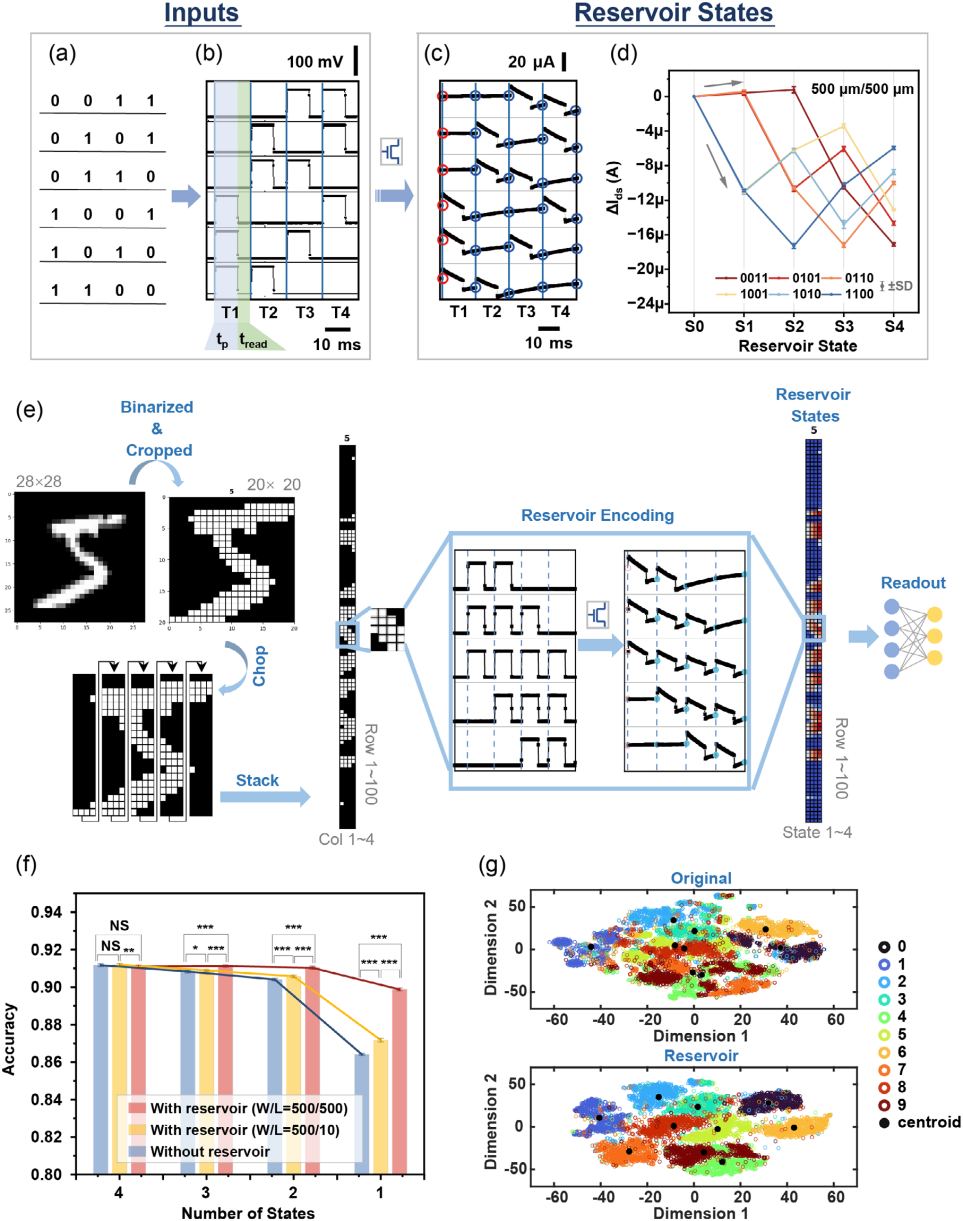
Temporal signal encoding through reservoir device and RC for classification of digit images. a) Combinations of 4‐bit binary digits containing two “0” and two “1” that occur in different sequences. b) Converted voltage pulse streams (timeframe = 16 ms, *t*_p_ = 10 ms, *V*_p_ = 0.1 V) and c) corresponding *I*_ds_ response of the memory OECT (*W* = 500 μm, *L* = 500 μm) read by *V*_ds_ at −0.2 V. d) Extracted reservoir states at each end of the timeframe on *I*_ds_ curves as normalized to the start point current. e) Preprocessing of images in handwritten digit MNIST dataset before encoding through the reservoir, and corresponding reservoir states. The original 28 × 28 pixel images are binarized and cropped to 20 × 20 and further chopped and stacked to a 100 × 4 column. The reshaped patterns are converted into pulse streams as input of the reservoir. f) Performance of the MNIST dataset classification through reservoirs or without reservoir encoding dependent on number of states. The best feature with the highest average accuracy among all combinations upon each number of states is reported. The results of hypothesis testing between groups are categorized by: NS: *p* > 0.05; *: *p* ≤ 0.05; **: *p* ≤ 0.01;***: *p* ≤ 0.001 (*n* = 10). g) 2D embedding of *t*‐SNE of the dataset without and with the encoding of the reservoir (500 μm/500 μm device, 1 state).

A key feature of RC is the dynamic neurons in the reservoir unit which possess fading memory and nonlinearly projecting properties. To configure the memory OECTs as physical reservoirs for RC, we operated the device within the STM region by applying short‐ and low‐voltage pulses on the gate terminal, meanwhile setting a proper timeframe of inputs on demands according to the forgetting time *τ*_relax_ of the device. In this way, the information of interest can be preprocessed and encoded into voltage pulse streams and fed into the reservoir. We applied a group of simple temporal inputs to test the reservoir device and demonstrated its repeatability and separability. Sixteen combinations of 4‐bit binary digits “0” and “1” (Figure S5, Supporting Information) were converted into square wave voltage pulse streams as the temporal inputs. To illustrate the encoding flow, six 4‐bit sequences that each contains two “1” and two “0” were arranged in different sequences and their corresponding pulse streams are shown in **Figure** **2**a,b. A programming pulse is assigned for “1” whereas the bias is kept at 0 V for “0”. The reading bias is set to 0 V all the time. The programming *V*_p_ and *t*_p_ were set at 100 mV and 10 ms to ensure STM operation while keeping a low‐energy and fast operation. Each bit corresponds to one timeframe that consists of 10 ms programming bias (*t*_p_) followed by 6 ms reading bias (*t*_read_). The four time frames of each stream are denoted as T1, T2, T3, and T4, respectively. The acquired *I*_ds_ under –0.2 V is shown in Figure 2c, and each downward/upward current changes match with the rising/falling edges of an input voltage pulse. The snapshot current value at each end of the time frame (S1, S2, S3, and S4) (highlighted by blue circles) were read. The corresponding current changes (Δ*I*_ds_) while comparing with the starting point (highlighted by a red circle) are defined as the reservoir states and they are summarized in Figure 2d. The reservoir states of the two devices (*W*/*L* = 500 μm/500 μm and 500 μm/10 μm) given by the same group of inputs are compared in Figure S6, Supporting Information. The mean standard deviation of extracted reservoir states from repeated measurements is 0.204 μA (*n* = 5), confirming their repeatability, while the distinguishable reservoir states given different inputs confirm the separability. As the 6 ms *t*_read_ was set close to the *τ*_relax_ of the 500 μm/10 μm device and significantly smaller than the *τ*_relax_ of the 500 μm/500 μm device (*τ*_relax_ = 35 ms), the accumulation of Δ*I*_ds_ given by adjacent programming pulses would be stronger in the later longer channel device and show more distinguishable reservoir states. These findings confirmed the importance of controlling the relationship between the timeframe setting and transient dynamics of the device and provided a way to improve the diversity of reservoir devices.

To further evaluate the capacity of the reservoir system, we conducted the benchmarking task of classification on the Modified National Institute of Standards and Technology (MNIST) dataset which contains 28 × 28 pixel images of handwritten digits^[^ ^39^ ^]^ based on the experimental reservoir states of 4‐bit full combinations (see Experimental Section). The images in the dataset were binarized and reshaped into 100 by 4 arrays, as the example of digit “5” shown in the flow in Figure 2e, so that each row can be converted into one encoding pulse stream that matches one of the 4‐bit combinations. The reservoir states acquired on devices in the 4‐bit binary digits experiment were matched to corresponding pulse streams to simulate the reservoir response for the whole MNIST dataset. This simulated reservoir generates *n* (1 ≤ *n* ≤ 4) reservoir states along each encoding stream, thereby an *n* × 100 feature matrix is settled for each sample, and then those features will be flattened as an input vector to the readout layer for decoding. Followed by training in the readout layer, the classification performance over the number of reservoir states *n* sent to the readout is shown in Figure 2f. All the combinations of reservoir states at each *n* were considered and the accuracy of the best feature is shown in the figure. For comparison, the original binarized patterns were also sent to the readout layer for classification without processing through the reservoir. The accuracy of the nonreservoir network drops more significantly when shrinking *n* from 4 to 1 while our system with 500 μm/500 μm reservoir device can still maintain around 90% (the comparisons are evidenced by *t*‐test, see asterisk brackets in Figure 2f). This ablation analysis indicates the benefit of the reservoir system in lowering the network complexity (the number of parameters to be trained decreases fourfold) and releasing computation power. However, the 500 μm/10 μm reservoir barely shows an obvious performance elevation compared to the case without the reservoir due to the mismatch between the *t*_read_ setting and its device dynamics. It suggests the importance of timeframe matching for effective reservoir encoding. The features that output from the 500 μm/500 μm reservoir and selected from the original dataset when the number of states is 1 are visualized by *t*‐distributed stochastic neighbor embedding (*t*‐SNE) for comparison (Figure 2g). Compared to the original data, the embedding of reservoir states shows a larger distance between clusters and a smaller distance within clusters, which reveals the significant effect of the reservoir on state separation (Figure S7, Supporting Information).

### 2.4 Artificial Synapse Based on LTM States of the Memory OECT

**Figure 3:**
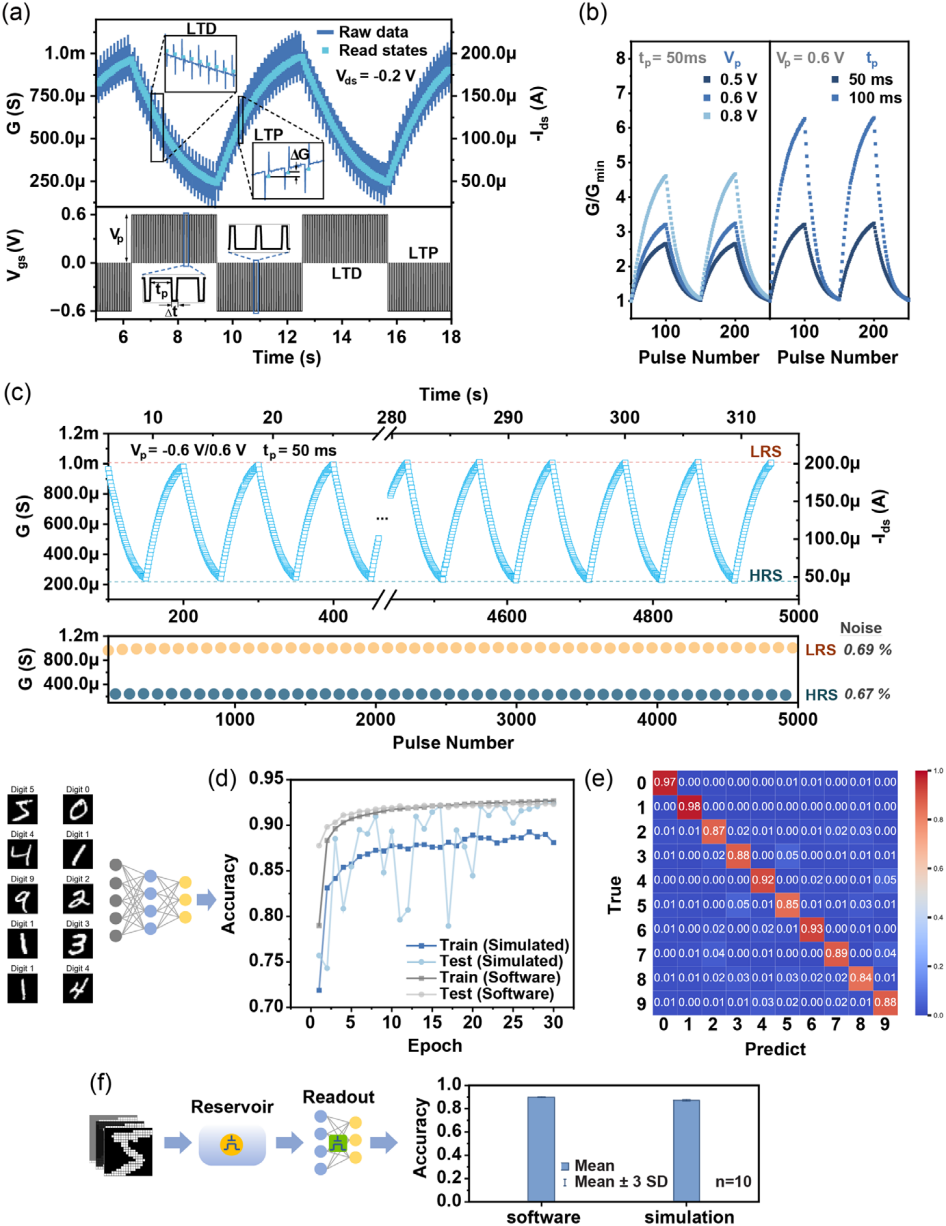
Synaptic behavior and neural network performance of the memory OECT device operated under LTM mode. a) Channel conductance response and corresponding square wave voltage pulse train input to the gate (*V*_p_ = 0.6 V, *t*_p_ = 50 ms, Δ*t* = 12.5 ms) for demonstration of LTD/LTP manipulation (device: *W* = 500 μm, *L* = 500 μm). The deep blue curve in the upper panel is raw recording and the pale blue scatter is read values extracted at each end of the pulse interval. b) The influence of *t*_p_ and *V*_p_ on LTD/LTP modulation. c) Extracted read states of LTD/LTP cycling for 50 cycles. The stability of the cycling is indicated by the standard deviation of maximum and minimum conductance across the cycling as denoted by LRS and HRS. d) Training and testing accuracy evolution of MINST dataset classification on a neural network using experimental synaptic weight update of the device (simulated) versus software weight update. The curves of one run out of ten repeated processes are shown. The first ten images in the dataset are attached aside. e) Confusion matrix for testing (simulated) of the MINST classification after 30 epochs (accuracy = 92.4%). f) Illustration of applying the synaptic device to the readout layer of a RC system in comparison with software‐conducted readout.

In biological neural networks, the chemical synapse mainly works for learning and memorizing by potentiating or depressing the synaptic plasticity according to the input information. Artificial synapses imitate biological systems by modulating the conductance state and postsynaptic current (PSC) in the active layer by presynaptic signal inputs. Herein, instead of using STM, the LTM property of the memory OECT was enabled by proper electrical configuration to perform the artificial synapse function. The long‐term depression–long‐term potentiation (LTD–LTP) cycling is conducted by applying successive positive and negative pulse trains on the gate while keeping constant *V*_ds_ at −0.2 V to read PSC simultaneously (**Figure** **3**a). Each input pulse results in a conductance change Δ*G*, which is considered as a weight update. The consequent modulation is the combined effects of pulse parameters such as *V*_p_, *t*_p_, device dimensions, semiconductor properties, and so on. Here, we use the device with *W*/*L* of 500 μm/500 μm to implement the LTD–LTP conductance modulation, where we further explored the pulse parameters based on the memory level map shown in Figure 1g. Under the condition of 0.5 V, 50 ms, the device is operating at the early transition from STM to LTM and it would preserve large room for further conductance (*G*) update, i.e., the device will not saturate too early. While we increase *V*_p_ from 0.5 to 0.8 V (maintain *t*_p_ = 50 ms), the *G*/*G*_minimum_ increases by 1.7‐fold (left panel of Figure 3b). If we increase *t*_p_ from 50 to 100 ms and keep *V*_p_ at 0.6 V, the *G*/*G*_minimum_ would increase by around twofold (right panel of Figure 3b). Considering the trade‐off between high performance and energy consumption, a moderate condition was selected (*V*_p_ = 0.6 V, *t*_p_ = 50 ms). Figure 3c shows repeated modulation cycles with 50 analog LTD and LTP states; the conductance shows a dynamic range (*G*_max_/*G*_min_) over fourfold. The noise, i.e., standard deviation/conductance range, of maximum conductance (low resistance state, LRS) and minimum conductance (high resistance state, HRS) is 0.69% and 0.67%, respectively, showing the low stochasticity of the cycling. The linearity and symmetry of the modulation are decent as the fitting results show in Figure S8a, Supporting Information. A statistic distribution of Δ*G* from about 5000 switching events is further analyzed by cumulative distribution function as shown in the heatmap in Figure S8b, Supporting Information, which suggests the great reproducibility of the weight update (i.e., a narrow Δ*G* distribution).

A two‐layer feedforward neural network was simulated based on the synaptic device for the benchmarking task of classification on the MNIST dataset of handwritten digits.^[^ ^39^ ^]^ The synaptic weight in the network is mapped with experimentally measured data which is extracted from 1000 analog conductance states of the LTP/LTD cycling (see Experimental Section). The evolution of network weights was initialized as random and updated based on the conductance update data via the backpropagation algorithm (Figure S9, Supporting Information), and the classification accuracy on the test set reached 91.1% ± 1.0% (*n* = 10) over 30 epochs, achieving a similar performance with the pure software implementation (91.7% ± 0.2%, *n* = 10) (Figure 3d). The confusion matrix of our LTM device‐based system on the test set is presented in Figure 3e. We then transferred this synaptic hardware to the neural network in the readout layer of a RC system (Figure 3f). Incorporating with the reservoir devices that deal with the MNIST dataset as discussed in Section 2.3, the simulated hardware readout can realize the recognition at a successful rate close to the ideal software readout. This demonstration proves the concept of a full‐OECT RC system for implementing AI tasks.

### 2.5 EMG Decoding with Full‐OECT Reservoir Computing Hardware

**Figure 4:**
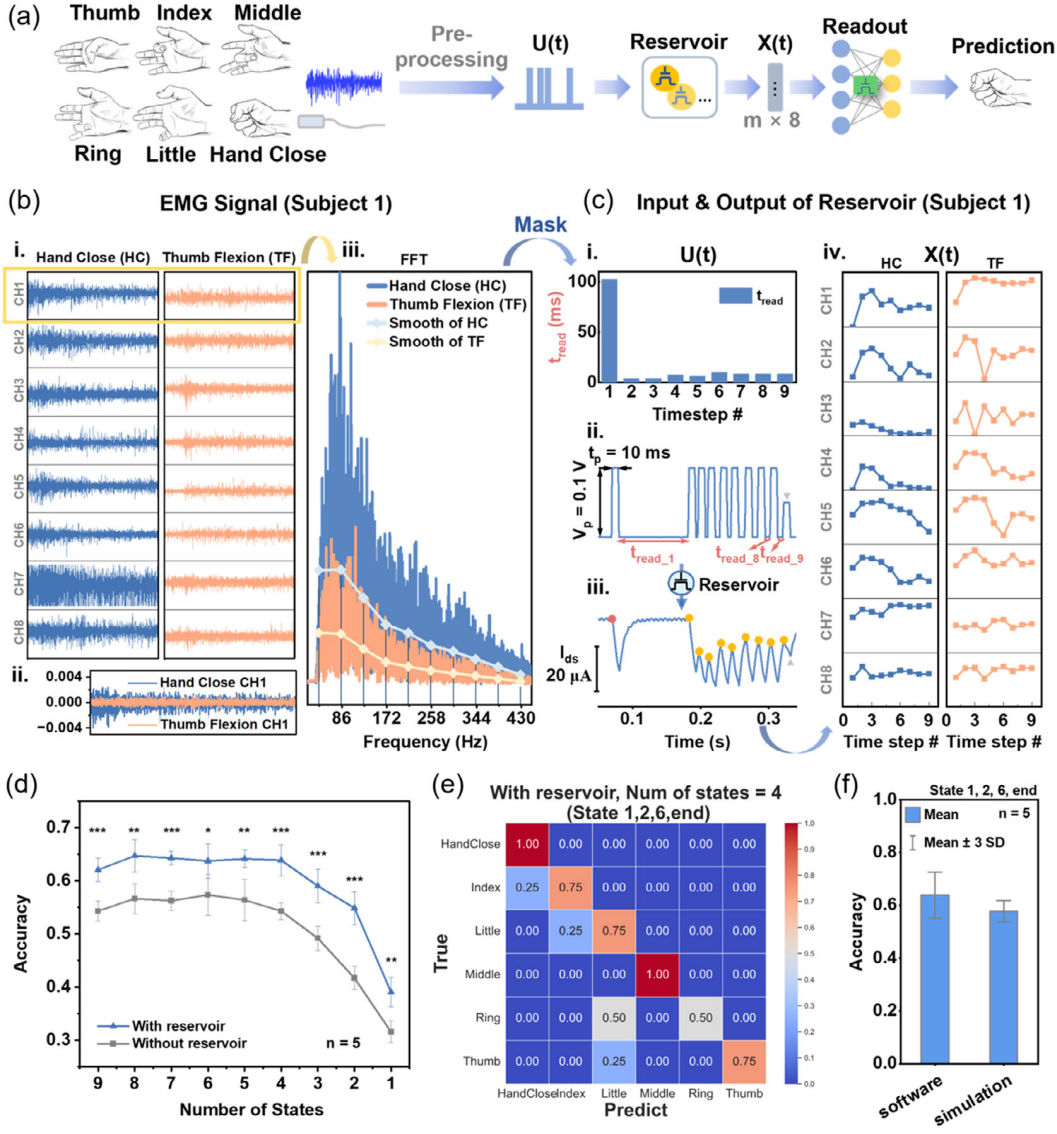
EMG decoding through RC system. a) Hand gestures to be classified and the processing flow of EMG signal decoding. b) Illustration of EMG signal presented in time and frequency domain (trial 1 of subject 1 in the dataset) with example hand movements. c) Illustration of (i) the output value of mathematic masking and (ii–iii) corresponding reservoir encoding process. The formed pulse stream carries the EMG information except the last pulse labeled with a gray triangle which is an ending mark. (iv) Reservoir states that correspond to samples shown in (b). The *y*‐axis ranges are unified. d) Performance on the EMG signal classification with/without encoding of the reservoir as the variable of the number of states. The scores shown are from the best combinations of reservoir states. The results of hypothesis testing between groups are categorized by: NS: *p* > 0.05; *: *p* ≤ 0.05; **: *p* ≤ 0.01;***: *p* ≤ 0.001 (*n* = 5). e) Confusion matrix of the decoding using the reservoir states read at time steps 1, 2, 6, 9 on the 500 μm/500 μm device (accuracy = 79.2%). f) Performance of the RC system with simulated weight update in the readout layer as compared with pure software weight update. The reservoir states were taken from device data (the 500 μm/500 μm device, state 1, 2, 6, 9), and the weight update is based on experimental data of the memory device as presented in Figure 3c.

Based on the success of temporal signal processing of the RC system, we further attempt to decode the EMG signal by our memory OECTs. EMG data were obtained from the open source dataset Electromyogram Repository,^[^ ^40^ ^]^ from which signal of six classes of hand gestures acquired on eight subjects by an 8‐channel EMG system was extracted and processed by our RC system, as illustrated in **Figure** **4**a. It is important to note that the raw EMG signals are preprocessed and converted into pulse streams ***U*** (*t*), and then analyzed through the RC system to generate the prediction of the hand gesture. The flow of preprocessing and reservoir encoding is illustrated in Figure 4b,c with example data of hand close and thumb flexion movements among the six gestures. First, the preprocessing transforms the time domain EMG signal (Figure 4b, panels i and ii) into the frequency domain by fast‐Fourier transform (FFT) (Figure 4b, panel iii), and the FFT spectra are smoothed at an averaging window (centers of windows are indicated by vertical lines). To convert features of the frequency spectra, including distribution and intensity, to pulse parameter variables (a group of *t*_read_ in this case), the averaged value in each window is mapped through the mathematical mask. As shown in Figure 4c, panel ii, the *t*_read_ is defined as the duration waited between the pulse voltage is applied where the current is measured. The mathematical mask converts the intensity difference between regions of the FFT spectra into *t*_read_ in a reciprocal way, i.e., if the change of mean intensity between adjacent regions is gentle, the *t*_read_ would be set long (see *t*_read_1_ in Figure 4c, panels i and ii as example) so that the reservoir response could be mild to reflect the gentle FFT spectra slope, and vice versa. The target *t*_read_ range for encoding was designed based on the *τ*_relax_ of the device as discussed in Note S2, Supporting Information. The detailed methodology of preprocessing can be found in the Experimental Section and Figure S10, Supporting Information. In this way, the input pulse streams of the reservoir device are constructed with timeframes and each timeframe contains a pulse duration time (*t*_p_) plus a *t*_read_ and takes one time step (Figure 4c ii.). The *V*_p_ and *t*_p_ of the pulse are fixed at 0.1 V and 10 ms to set the OECT in the STM regime while remaining *t*_read_ as a variable for information encoding. Then, the reservoir states ***X*** (*t*) are extracted from the *I*_ds_ response of the device (500 μm/500 μm) as read at the end of time steps (yellow spots in Figure 4c, panel iii) versus the start point (red spot), which forms the feature pattern of each EMG channel for each sample (Figure 4c, panel iv). Afterward, the *m* selected reservoir states (1 ≤ *m* ≤ 9) from all the eight streams (corresponding to eight EMG channels) were raveled as one input vector (feature size = *m* × 8) for a sample and sent to the readout layer for decoding (Figure 4a).

In the readout layer, a one‐layer perceptron was used for classification, where sixfold cross‐validation was employed for fair evaluation, and the average score of five repeats of the cross‐validation was recorded (see Experimental Section). Ablation analysis was carried out regarding the number of reservoir states *m* selected for classification to find a minimized network size with adequate performance. The effect of adding combinations of 1st–8th states to the end (9th) state was considered (Note S3, Supporting Information), and the highest score (best feature selection) upon each number of states was presented in the results (Figure 4d). Specific scores of all the combinations and their distributions are shown in Figure S11, Supporting Information, and the names of the best combinations are shown in Table S1, Supporting Information. Encoded through the reservoir devices, a recognition accuracy above 60% was reached when *m* = 9, and this level can be kept if shrinking the number of nodes to *m* = 4. The input vector (***U*** (*t*)) of the reservoir was also fed to the readout layer directly for classification bypassing the reservoir, compared to which the overall performances of reservoirs are significantly higher (see asterisks for *t*‐test evaluation in Figure 4d). This proves the effectiveness of reservoir encoding in this case for the EMG signal decoding task. The confusion matrix of the best shot (*m* = 4, 79.2%) is shown in Figure 4e. In this way, the number of parameters to be trained to realize this task is 192 (4 states × 8 channels × 6 outputs), which is largely downsized compared to using the raw EMG data (20 000 data points per channel for 5 s recording). One should note that the limited sample number in the dataset (24 samples for each gesture) and the disturbance of inter‐ and intrasubject variations may potentially limit the successful rate of classification, which can be found in the distribution of the encoded data (Figure S12, Supporting Information) and discussed in Note S2, Supporting Information.

To demonstrate full‐OECT implementation of EMG decoding by RC, similar to the previous MNIST task, we simulated the in situ training of the OECT‐based readout layer according to experimentally measured LTM for hand gesture recognition (see Methods, Supporting Information). The analog conductance update of the LTM device used is the same as in MNIST tasks. The classification based on experimental reservoir states and simulation hardware readout layer can maintain 91% of the results conducted on the software readout layer (Figure 4f). In short, we can configure the same memory OECT to either a reservoir neuron or artificial synapse and integrate them as a whole RC system for specific biological signal decoding tasks. This demonstrates the potential of constructing RC hardware with full‐OECT key components generated from one‐batch fabrication, which would reduce the hardware complexity and provide a promising application prospect such as edge computing chips for biosignal decoding.

## 3 Conclusions

In conclusion, this work provides insights into the application of memory OECT with PEDOT:Tos/PTHF active layer for neuromorphic computing. Investigations on the memory level and relaxation time of the device established foundational guidance for flexible electrical configuration by setting pulse voltage *V*_p_ and pulse duration *t*_p_, enabling the switching of the device between STM and LTM functions. Based on these, this dual‐modal memory OECT device was configured as both artificial neuron and artificial synapse to perform reservoir encoding and weight updating in RC. The performance of each function was proved with classification accuracy above 90% on the benchmarking MNIST dataset. Combining the reservoir and synaptic device counterparts, a full‐OECT RC system was constructed and passed the evaluation of the MNIST classification task. The capability and simplicity of this full‐OECT RC system on biological signal decoding were finally demonstrated with successful hand gesture recognition from EMG signals at a reduced network size and low hardware complexity. Those results implied the potential of homogeneous integration of the dual‐modal memory OECT to construct neuromorphic computing hardware for decoding complex signals and solving real‐life problems. Further improvements could be expected such as upgrading the device performance by precise tailoring and optimized control, adapting the device to a variety of biological signals, and conducting decrypt for human–machine interface applications.

## 4 Experimental Section

4.1

4.1.1

##### Fabrication of Memory OECT

The PEDOT:Tos/PTHF‐based memory OECTs were fabricated referring to the previously reported procedures.^[^ ^38^ ^]^ Briefly, Cr/Au (5 nm/50 nm) source and drain electrodes were thermal evaporated onto photoresist‐patterned glass substrates, after which two parylene layers for encapsulation and sacrifice were deposited and patterned to present channel areas. After spin coating of the specifically formulated precursor solution, the channel layer was formed by vapor phase polymerization in a tube furnace under 80 °C for 1 h and the yield device was rinsed with deionized water for 1 min. The percentage of PTHF in the composition is controlled by tuning the weight of PTHF added into the precursor while maintaining the mass of Fe(Tos)_3_ the same. Afterward, a 3D‐printed reservoir was bonded onto the top of the device via thermal crosslinking of PDMS (Sylgard 184 clear) to allow the electrolyte solution to fill in. An Ag/AgCl electrochemical reference electrode was hanging into the electrolyte as the gate electrode and the reservoir was filled with 0.1 m NaCl aqueous as electrolyte.

##### Electrical Characterization

The source electrode of the device was grounded, and measurement voltages were applied between electrodes. Transfer curves were measured by Keithley 2636B source meter. The input signals of bipolar pulse stimulation and reservoir encoding were supplied by a programmable power supplier (PBZ40‐10, KIKUSUI). In practice, all pulse streams were input to one reservoir device under test in a way of time‐division multiplexing. For conductance modulation of the synaptic device, the voltage pulse train applied to the gate was generated by two Agilent 33210 arbitrary function generators alternatively. To record the source–drain current output, a DMM 6500 digital multimeter was connected in series with the channel and set in digital current mode (sampling rate of 5 kHz for bipolar pulse measurement and 1 kHz for reservoir state and conductance modulation measurements) while one output channel of Keithley 2636B source meter was used to supply the source‐drain voltage constantly at −0.2 V. All the operation of equipment mentioned above was in the charge of customed LabVIEW programs.

##### Time Constant Estimation

The resulting channel current was fitted by a two‐phase exponential function operated in OriginLAB, from which two time constants *τ*_1_ and *τ*_2_ corresponding to the two phases were obtained, and a weighted time constant *τ* was calculated by $\tau =w_{1}\times \tau_{1}+w_{2}\times \tau_{2}$, where $w_{1}=\frac{A_{1}}{A_{1}+A_{2}}$ and $w_{2}=1-w_{1}$.

##### Artificial Neural Network and Reservoir Computing Simulations

To perform the neural network, the weights were linearly mapped from experimental conductance modulation data of the 500 μm/500 μm device driven by ±0.6 V 50 ms pulse train, where the mapping gradients were optimized, and backpropagation algorithm was applied for weight updating. In the simulation of the reservoir for RC, the reservoir states were mapped from the device responses to the pulse streams that were encoded from 4‐bit binary digits and matched to the pretreated images from the MNIST dataset which were transformed into 20 rows of 4‐bit binary digits. The readout layer of the RC system was performed based on both software calculations and hardware simulations. Details about the dataset preprocessing, network structures, hyperparameters and activation functions, and train/test dataset distributions are described in Methods, Supporting Information.

##### EMG Signal Preprocessing

EMG data were extracted from the Electromyogram Repository.^[^ ^40^ ^]^ All the preprocessing was conducted with MATLAB R2021b. The dataset contains a signal of three trials of 15 classes of finger movements recorded on eight subjects by an 8‐channel EMG system at a 4 kHz sampling rate. The first 5 s of each recording of six classes from the dataset (hand close, and flexion of index, little, middle, ring, and thumb) were used for classification in this work. The raw data in the time domain were bandpass filtered between 20 and 450 Hz (fourth‐order Butterworth bandpass filter) with a notch filter at 50 Hz (second‐order Butterworth bandstop filter), and FFT was applied to convert data into the frequency domain. The FFT spectra within 20–450 Hz were sliced into ten regions and the mean *y* value of each region was calculated. Mathematical mask: Absolute differences between mean values of adjacent regions were calculated and the reciprocal of those differences were taken. This left 9 reciprocal values for the signal of each channel and the whole set results of 8 channels in each sample were linearly projected onto a target time range as *t*_read_ (3–105 ms was used here) in the programming pulse streams. The *V*_p_ and *t*_p_ in the pulse streams were fixed at 0.1 V and 10 ms. The method is further elaborated in Note S2, Supporting Information.

## Conflict of Interest

The authors declare no conflict of interest.

## Author Contributions

**Yuyang Yin** and **Paddy Kwok Leung Chan** conceived and oversaw the project. **Yuyang Yin** fabricated the devices, formed customized programs and setups for electrical measurements, and conducted device tests and data pre‐ and postprocessing. **Shaocong Wang** and **Zhongrui Wang** designed the neural network simulations and provided consultations on model optimization and results analyses. **Yuyang Yin** and **Ruihong Weng** modified the code of neural network simulations to fit specific tasks. **Yuyang Yin** and **Paddy Kwok Leung Chan** designed the reservoir simulation. **Yuyang Yin**, **Ruihong Weng**, and **Na Xiao** discussed the results of synaptic weight updating and reservoir computing tasks, and **Ruihong Weng** conducted statistical analyses on computing results. **Yuyang Yin** and **Na Xiao** analyzed the results of electrical and electrochemical characterizations of the OECTs. **Yuyang Yin**, **Jianni Deng**, and **Paddy Kwok Leung Chan** conceived the scheme of EMG data preprocessing. **Yuyang Yin** and **Qian Wang** analyzed the dynamic properties of the device. **Yuyang Yin**, **Paddy Kwok Leung Chan**, **Na Xiao**, and **Ruihong Weng** wrote the manuscript. All authors discussed the results and contributed to the preparation and revisions of the manuscript.

## Supporting information

Supplementary Material

## Data Availability

The data that support the findings of this study are available from the corresponding author upon reasonable request.
