# Supplementary material for: A Dual‐Modal Memory Organic Electrochemical Transistor Implementation for Reservoir Computing
Source: Small Sci. 2024 Oct 16;5(1):2400415. doi: 10.1002/smsc.202400415 (PMC11935116; doi:10.1002/smsc.202400415)
Supplement: Supplementary file 1 — Supplementary Material [file SMSC-5-2400415-s001.pdf]

Supporting Information

A dual-modal memory organic electrochemical transistor (OECT) implementation for full-OECT reservoir computing

Yuyang Yin, Shaocong Wang, Ruihong Weng, Na Xiao, Jianni Deng, Qian Wang, Zhongrui Wang, Paddy K. L. Chan*

Table of Contents

- **Methods**
- **Figures and Tables**
- **Notes**

Methods

Synaptic weight mapping and artificial neural network simulation

A fully connected neural network with one hidden layer was used for classification. The network consists of 784 (28×28) input nodes, 50 hidden nodes, and 10 output nodes. 60000 samples were used as training set and 10000 were used for testing. The weights were linearly mapped from experimental conductance modulation data, where the mapping gradients were optimized. 10 cycles of potentiation and depression cycling on a 500 μm/500 μm device driven by ± 0.6 V 50 ms pulse train were used. A backpropagation algorithm was applied for weight optimization. Cross entropy was set as the loss function. Hyperparameters were tuned, and 30 epochs were run for training and testing.

4-bit pattern and handwritten digit image encoding through reservoir devices

In the experiment, 4-bit patterns from 16 possible combinations were encoded to a pulse train and mapped to 4 states by reservoir devices. The experiment was repeated 5 times and generated 16×4 ×5=320 states in total. For the handwritten digits, the original 28*28 images from the MNIST dataset were cropped to 20*20 pixels by discarding 4 elements from margins and chopped into 5 pieces along the vertical axis to make each piece contain 20 rows and 4 columns. These pieces of each sample were stacked over one another sequentially to form a new array with 100 rows and 4 columns. The data was scaled over 255 and binarized through a threshold of 0.1, so that each row of the new array can be represented by a 4-bit binary number. In the simulation, for each 4-bit row of the MNIST dataset samples, corresponding 4 reservoir states were randomly chosen from the 5 experimental results. Those reservoir states of each sample were flattened as a feature vector and passed to the readout layer for classification.

The readout layer of a reservoir computing system

A fully connected perceptron neural network without a hidden layer running based on a backpropagation algorithm was used for classification (Input node N = the number of features and output node M = the number of classes). In all cases, the input data array was normalized by scipy.stats.zscore function along the sample axis before being fed to the neural network. For MNIST dataset classification based on simulated reservoir encoded data, there are 60000 samples in the training set and 10000 samples in the testing set. SoftMax was chosen as the activation function. Before activation, the Dropout and ReLU function was used. Cross entropy was set as the loss function. SGD was used as optimizer. The training/testing process ran for 100 epochs. Average scores were recorded from ten repeats of the process. For the EMG decoding task, due to the limited sample number from the dataset (144 samples in total for 6 classes of hand gestures), six-fold cross-validation was employed for better generalization and hyperparameter tuning. The model at the 100th epoch was saved for each fold, and the average performance on the test set of six models was taken as the score. Statistical analysis was conducted on three repeated trials of the six-fold cross-validation. For both the MNIST dataset and EMG classification tasks, ablation analysis was performed with a decreasing number of features in the input vector of the readout layer. All combinations of features were considered, and the best features were chosen by evaluating the average validation set performance. For the simulated readout layer training, the algorithm of weight mapping from experimental data was modified based on the artificial neural network simulation part.

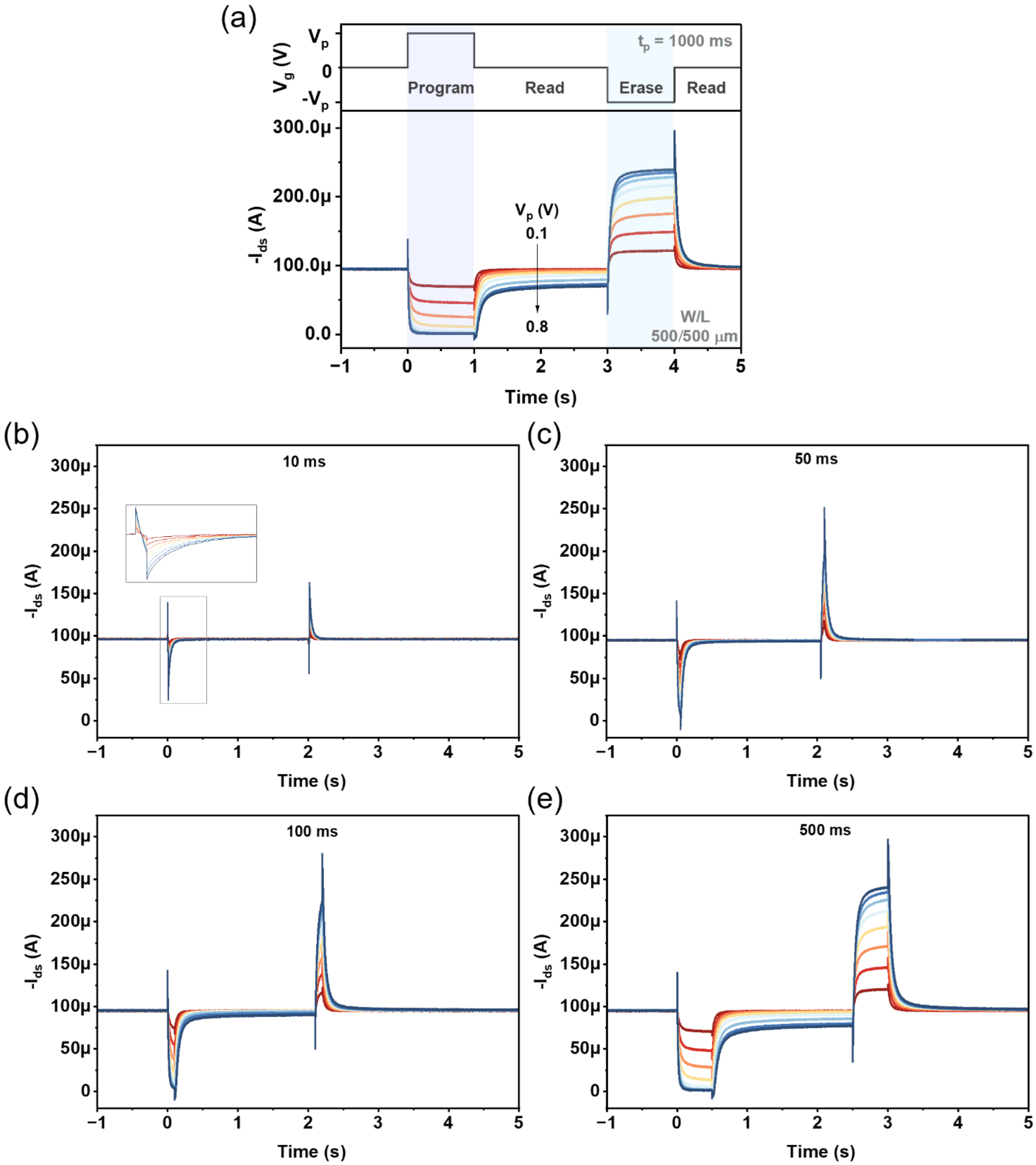

Figure S1. Channel current response of memory OECT (W = 500 µm, L = 500 µm) given by couples of bipolar voltage pulses with varied duration and amplitude.

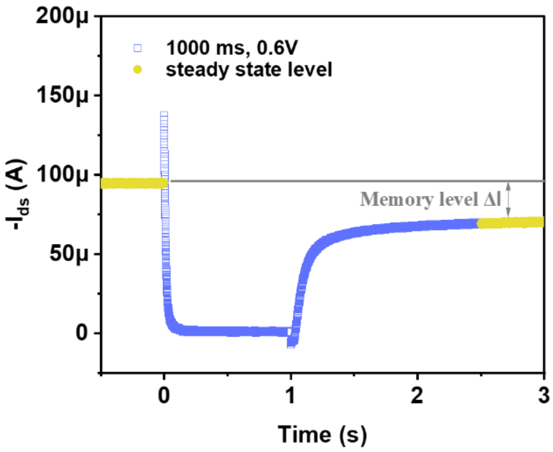

Figure S2. Memory level evaluation. Illustration of the method to extract memory levels on I_{ds} response curves.

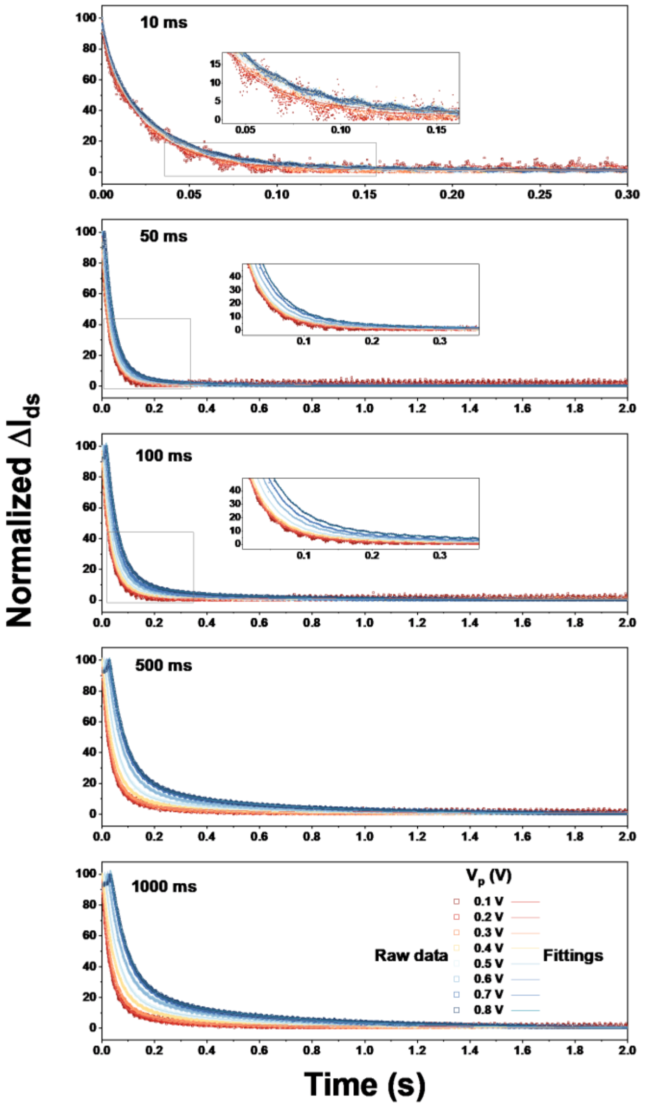

Figure S3. Exponential fittings on the relaxation side of I_{ds} curves given by positive-amplitude gate voltage pulses in Figure S1. Each group of curves is normalized to the range of 0 to 100. The mean and deviation of the R-square of all the fittings is 0.997±0.00252.

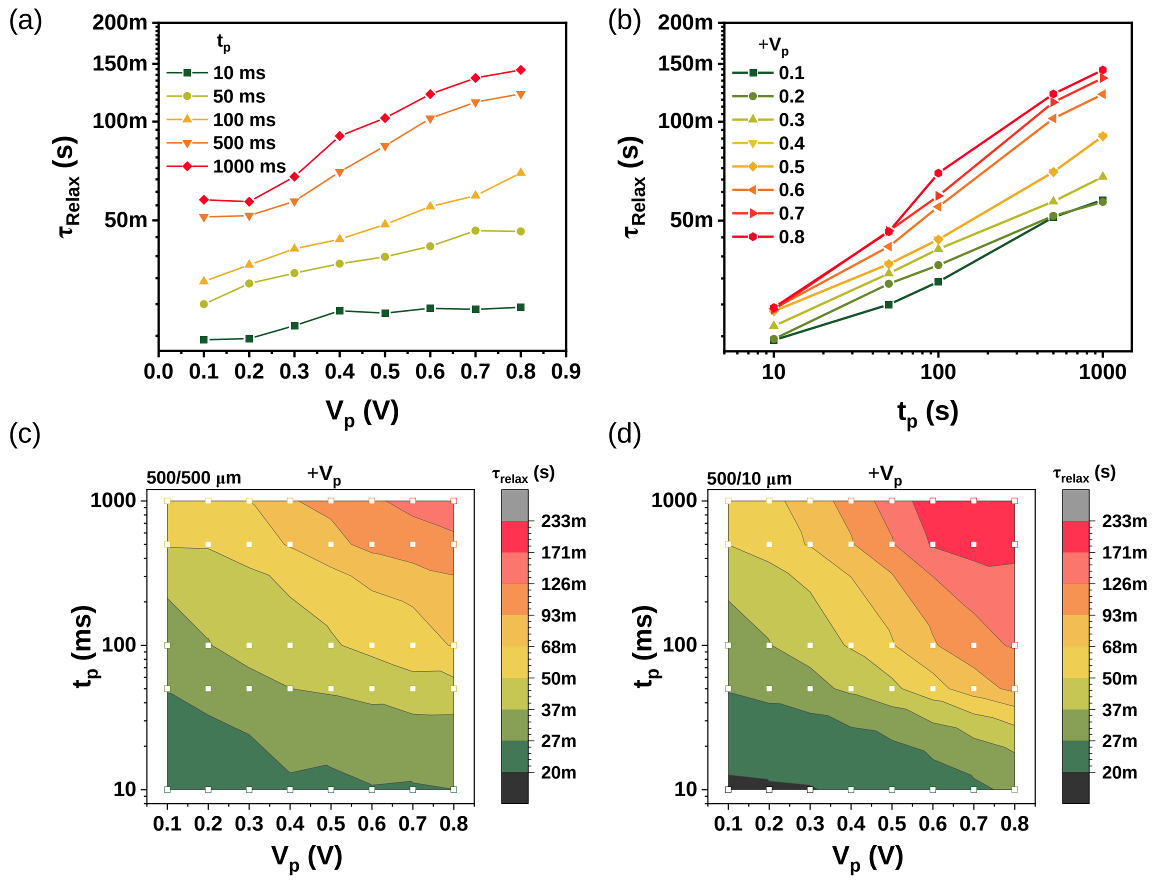

Figure S4. Dependencies of τ_{relax} on V_p and t_p of the stimulation voltage pulse. (a)-(b). τ_{relax} of 500 μm /500 μm device as a function of V_p and t_p. (c)-(d). Contour map of τ_{relax} of a 500 μm /500 μm device and a 500 μm /10 μm device given by positive gate voltage pulses, respectively.

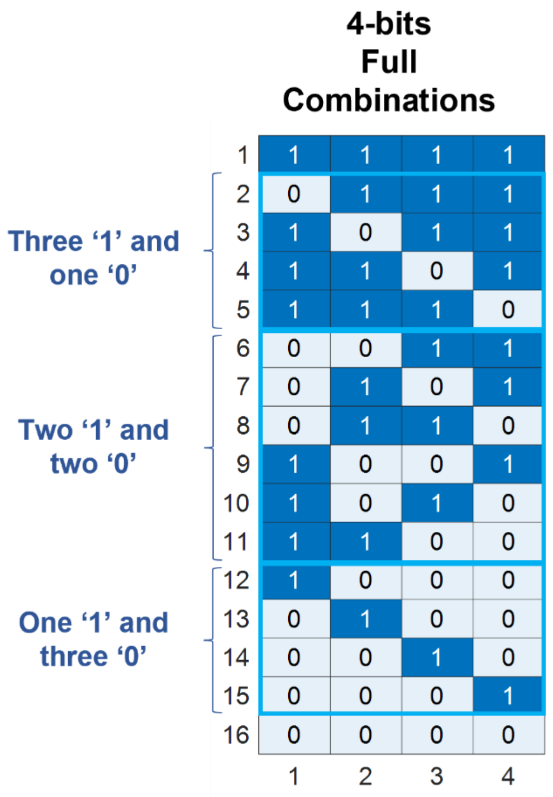

Figure S5. All combinations of 4-bit binary digits

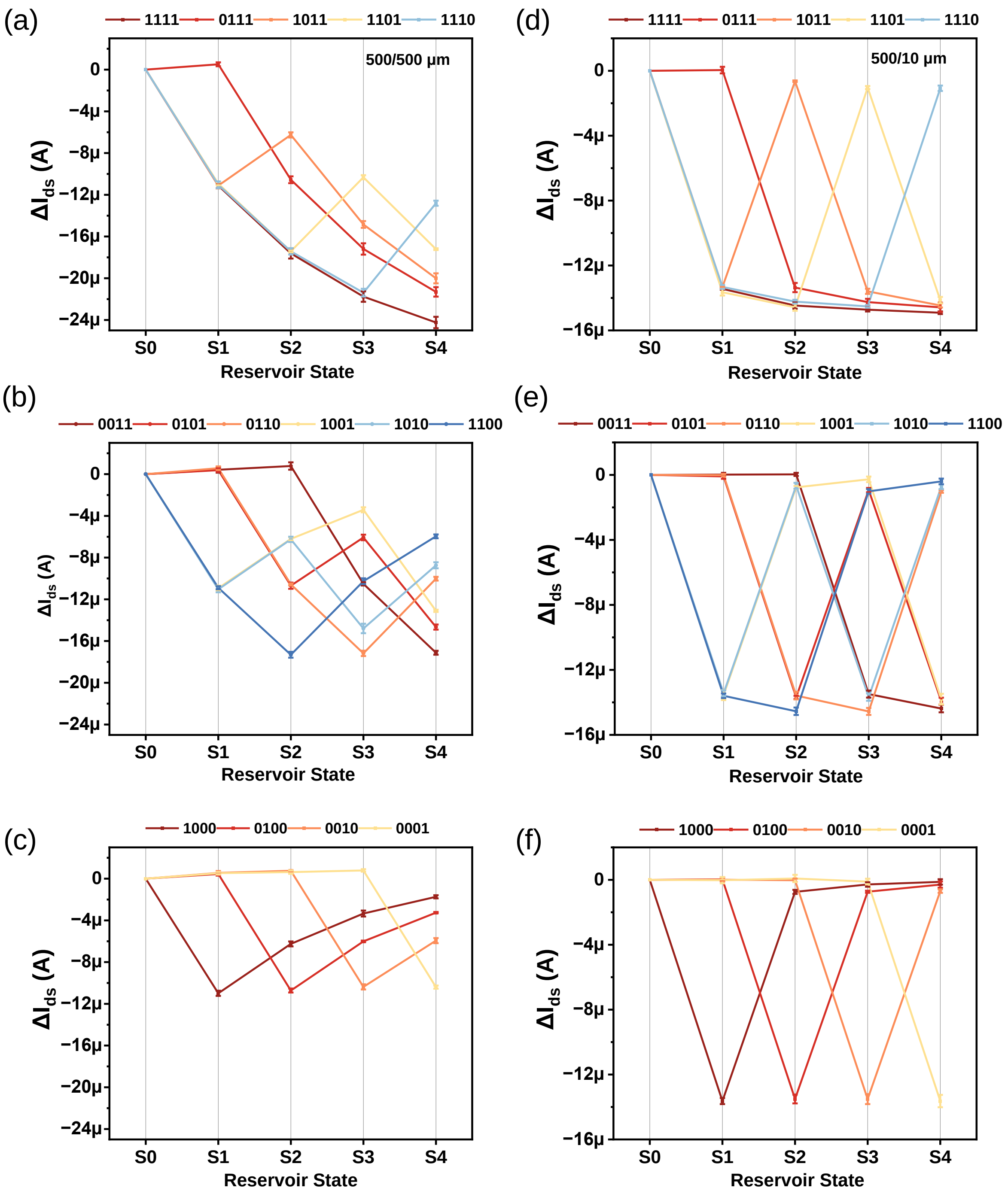

Figure S6. Reservoir states in response to 15 pulse streams converted from 4-bit binary digits. (a)-(c) Measure on large channel device (W = 500 μm, L = 500 μm). The average standard deviation is 0.240 μA. (d)-(f). Measured on a small channel device (W = 500 μm, L = 10 μm). The average standard deviation is 0.167 μA.

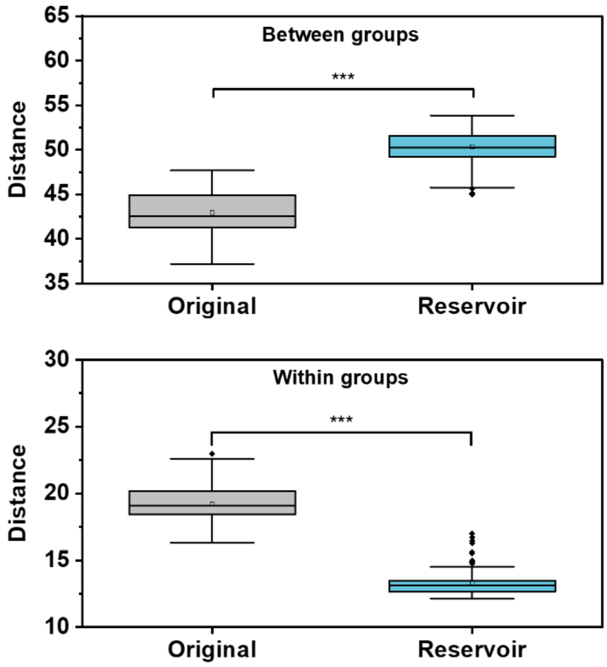

Figure S7. Distances between- and within- t-SNE embedding clusters of the original dataset and reservoir states output from the 500 µm/500 µm device (number of states n = 1). Hypothesis testing was validated with results of 100 repeats.

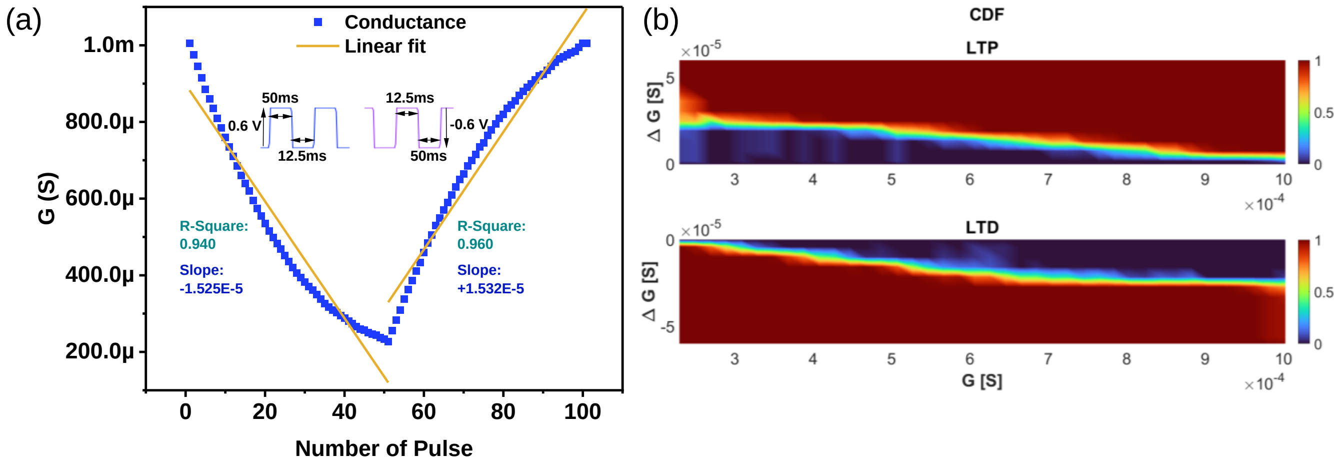

Figure S8. Evaluation of LTD/LTP modulation curves. (a) Linear fitting along LTD and LTP sides on the last cycle of the 50-cycling test. The R^2 of the regression shows the goodness of the fitting, and the similar slope of each side indicates great symmetry of the modulation. (b) The cumulative distribution function (CDF) heatmap shows the distribution of ΔG at each conductance state as extracted from the cycling test in Figure 3c, main text. The whole G range is divided into 40 windows while the ΔG range is broken into 20 windows for counting. The probability of ΔG under each G window is accumulated from ΔG = 0 to |ΔG| = 6.5×10^{-5} S to calculate the

cumulative distribution.

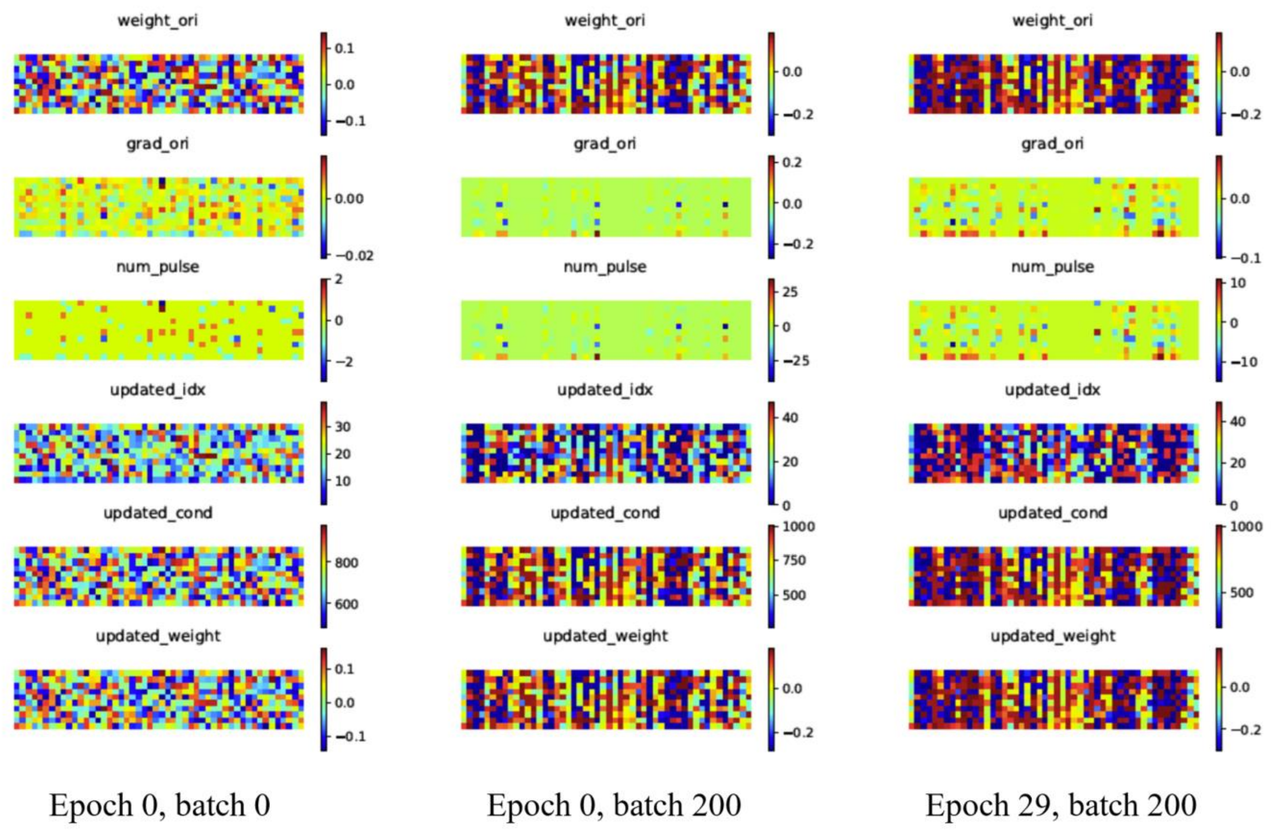

Figure S9. Visualization of synaptic weight update process during the training of the neural network. The evolution of the connectivity between the hidden layer and output layer is shown in the 50×10 matrix, and the colormap indicates the values. The weights on the software were initialized as random and then updated by mapping from the updated device conductance.

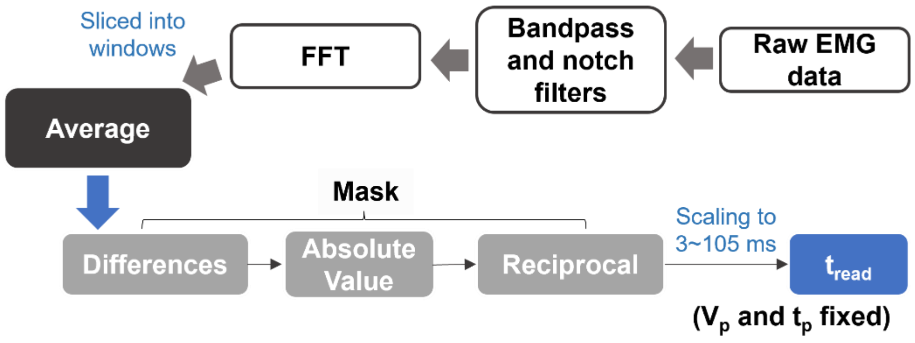

Figure S10. Flow chart of mathematical masking strategies for converting EMG signal to input pulse streams of the reservoir.

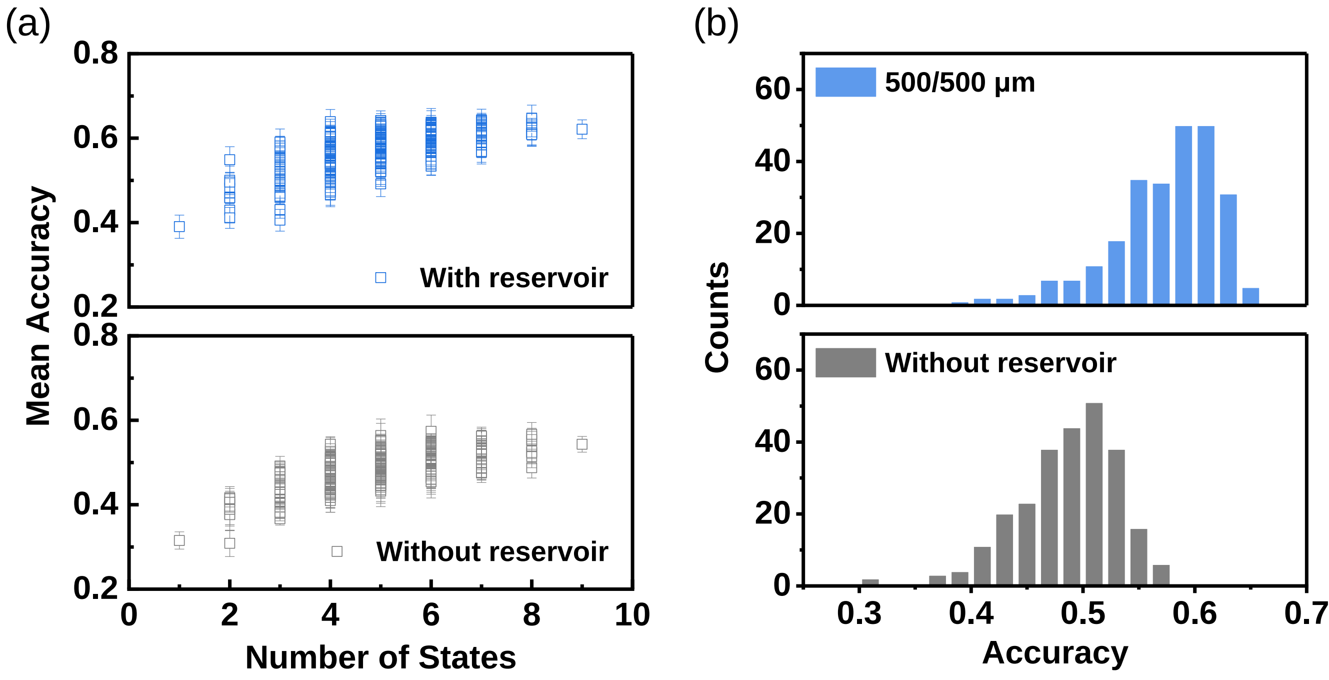

Figure S11. Scores of the EMG classification in all combinations of reservoir states in each case, and corresponding distributions. The error bar is from 5 repeats of the six-fold cross-validation.

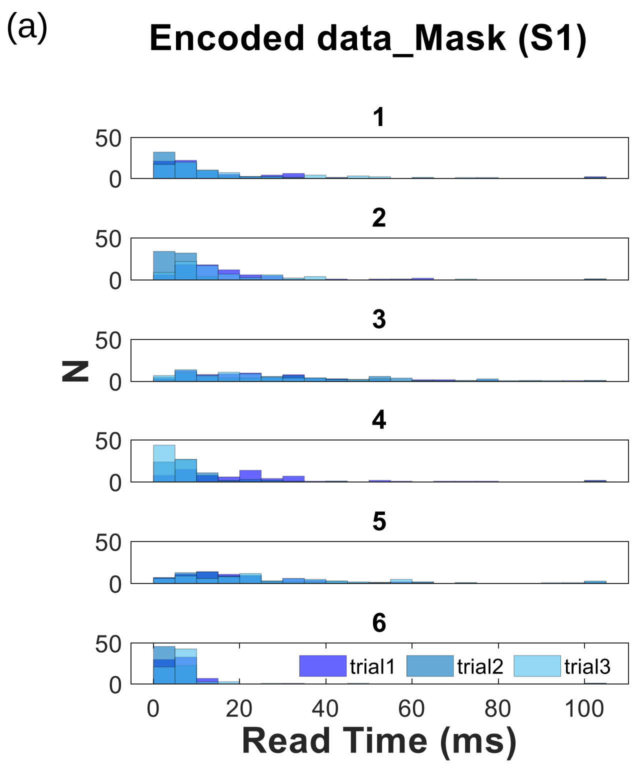

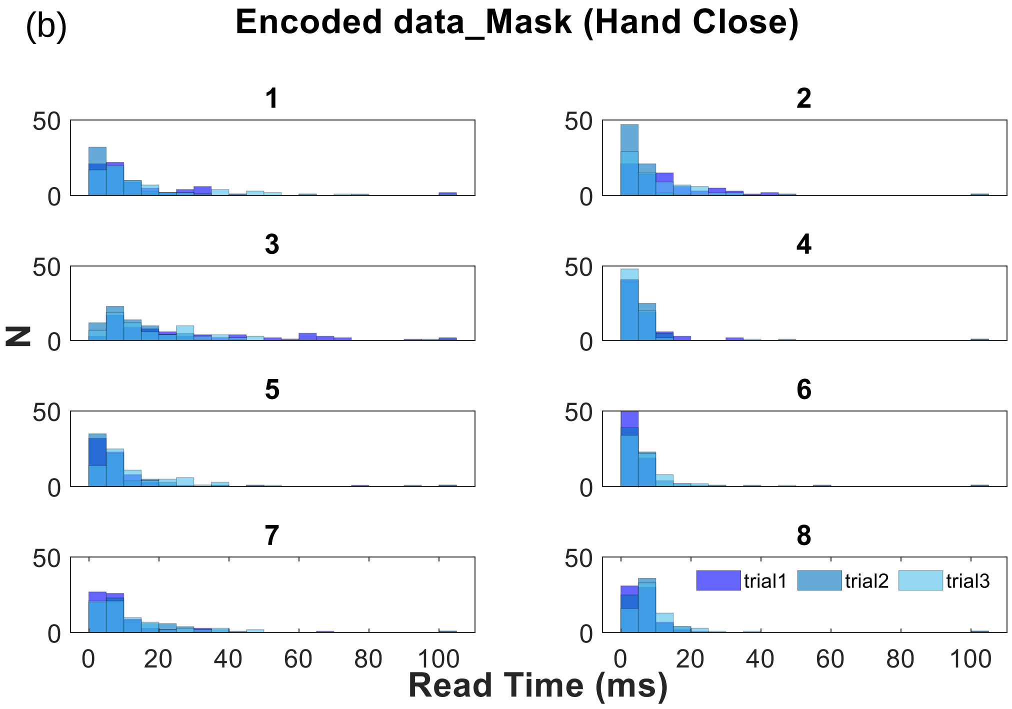

Figure S12. Histograms of the variable values converted from EMG signal via Mask. (a) As compared among six classes of hand movements (No.1~6 represents hand close and index, little, middle, ring, and thumb flexion. Data are Subject 1). (b) As compared among subjects (No.1~8 stands for Subject 1~8. Data are from Hand Close samples).

Table 1. Mean accuracies of the best combinations of each number of selected reservoir states. The combinations are indicated by the state number (# 1~9).

No. of States	Mask			
	Device 500/500		Without Reservoir	
	# Time step	Mean(Acc)	# Time step	Mean(Acc)
**1**	9	0.390	9	0.315
**2**	2,9	0.549	3,9	0.417
**3**	1,5,9	0.590	2,4,9	0.492
**4**	1,2,6,9	0.639	2,5,6,9	0.543
**5**	1,2,5,6,9	0.642	1,2,5,6,9	0.564
**6**	1,2,4,6,8,9	0.638	1,2,5,6,8,9	0.573
**7**	1,2,4,6,7,8,9	0.643	1,2,5,6,7,8,9	0.563
**8**	1,2,4,5,6,7,8,9	0.647	1,2,4,5,6,7,8,9	0.567
**9**	1~9	0.621	1~9	0.543

Notes

Note 1. Memory level and STM-LTM boundary definition

As presented in Figure S2, memory level values are extracted from the difference of steady state current before and after a gate stimulation applied. To avoid the influence of sampling noise, the steady state current value is represented by the mean of data within a 0.5 s window consisting of 2500 data point (at sampling rate 5000 Hz) as the highlight on plots shown. The contour map presents the dependency of relax time constants on V_p and t_p of gate pulse and the square scatters inside represent experimental measurements. The color code and contour lines are determined by interpolation over experimental data.

To present the degree of memory level of the device induced by single pulse in a compatible way, since the ON/OFF range of different device might influence the absolute value of memory level, we use relative percentage and define the memory level induced by 0.8 V 1000 ms pulse as 100 % because under this condition devices normally are totally switched (turned OFF or turned ON) and the caused memory level could be close to the upper limit. Base on this the lower limit for long-term memory is set at 0.5 %, providing a reference boundary between volatile and non-volatile regime.

Note 2. EMG signal encoding

The encoding of each EMG sample via **Mask** finally transfers extracted values into read time of pulse streams in range of 3 to 105 ms. The low limit is $0.5\times\tau_{relax}$ of the 500/10 μm device and the high limit is $3\times\tau_{relax}$ of the 500/500 μm device given by one 0.1 V 10 ms pulse (τ_{relax} = 6 ms and 35 ms for 500/10 μm and 500/500 μm devices employed in this experiment), aiming to cover the scale that enabling the two devices to show different response intensity. It should be note that it is possible to elevate the reservoir computing performance by further optimizing the value of

parameters mentioned above.

The histograms of typical encoded data are collected in Figure S12. For EMG data of six movements detected on one subject (S1), the distribution of encoded data via **Mask** is obviously different among those six classes, and the distributions are almost concentrated around 0 to 20 ms (Figure S12a). For signal of one certain movement (Hand close), encoded data of eight subjects are compared, showing the possible inter-subject difference (Figure S12b). Plus, three trials of one subject presented in colors are not overlapped well in some cases indicating the intra-subject error. These are intrinsic factors of the dataset in use that might disturb the decoding.

Note 3 Reservoir state combinations and ablation analysis in EMG decoding

Only using the reservoir state at the end time step along each encoding stream might lead to loss of information in early time steps. Introducing reservoir states at time steps other than the last step could improve this, similar to the concept of virtual nodes.[1] Not only the number of states but also which states are selected matters on the classification performance, because some states could carry more useful features of the input signal than others. To avoid the bias introduced by intentional selection, we do the evaluation on all combinations of reservoir states upon state number from 1 to M-1, where M is the total number of time steps which is 9 in this case (the number of combinations is 256).

References:

1. Moon, J.; Ma, W.; Shin, J. H.; Cai, F.; Du, C.; Lee, S. H.; Lu, W. D., Temporal data classification and forecasting using a memristor-based reservoir computing system. *Nature Electronics* **2019,** *2* (10), 480-487.
